# The Migrainous Brain: What You See Is Not All You Get?

**DOI:** 10.1371/journal.pmed.0030404

**Published:** 2006-10-17

**Authors:** Peter J Goadsby

## Abstract

Goadsby discusses a new study published in*PLoS Medicine* of the visual motion-processing network in migraine.

Migraine is an episodic brain disorder [1] that affects about 15 percent of the population [2,3]. The disorder can be highly disabling [4], and has been estimated to be the most costly neurological disorder in the European Community, costing more than €27 billion per year [5].

Two clinical forms are commonly seen: migraine with aura and migraine without aura [6]. Aura in this context is defined as a recurrent disorder manifesting in attacks of reversible focal neurological symptoms, usually developing gradually over a period of five to 20 minutes and lasting for less than 60 minutes. These symptoms are typically visual [7], and are often described as bright jagged lines (fortification spectra) that move across the visual field, often followed by visual loss (scotoma).

The phenomenon of migraine has been known since antiquity. Now there are new data on anatomical alterations in the visual motion-processing regions. Do these new data shed light on aura, or perhaps illuminate more basic principles about the migrainous brain? Is the traditional view—that the migrainous brain is structurally normal—incorrect?

## A New Study of the Visual Motion-Processing Network in Migraine

In a new study published in *PLoS Medicine*, Granziera and colleagues [8] used magnetic resonance imaging to perform high-resolution measurements of cortical thickness and diffusion tensor imaging to study the anatomy of the motion-processing network in patients with migraine and in healthy controls. The authors found significant differences between patients and controls in MT+ and V3A, both motion-processing visual cortical regions [9]. These differences were seen both in patients who experienced migraine with aura and patients who experienced migraine without aura.

These data need to be considered in the light of recent structural imaging suggesting, in a study of a random sample of patients with migraine, that those with aura may be particularly at risk for brain lesions on magnetic resonance imaging [10]. The data also need to be seen in the context of no detected change in voxel-based morphometry (a computational approach to neuroanatomy that measures differences in local concentrations of brain tissue) in the brains of patients with migraine [11]. However, voxel-based morphometry is probably not as sensitive as the technique used by Granziera and colleagues in their new study.

The phenomenon of migraine has been known since antiquity.

## Clinical Implications

The authors of the new study suggest that the findings may be used as a biomarker. Certainly the findings will not be useful as diagnostic tools, since they overlap with healthy controls, and it still remains simpler, and indeed the gold standard, to take a history from the patient. Moreover, it will be important to see whether the changes seen in Granziera and colleagues' study occur in other primary headaches.

It does seem likely that the findings relate to migraine given the motion sensitivity of migraineurs [12] and the very obvious *travel sickness* that younger migraineurs so often report. Indeed the literature on vertigo in migraine [13] further colours a landscape that suggests migraineurs have very distinct perceptual motion problems. For the clinician, one could take home the message from this new study that patients with migraine might be expected to present some often curious clinical features of motion sensitivity, and the new data give a strong biological context to an otherwise somewhat soft history.

## The Experimental Homologue of the Migraine Aura

Cortical spreading depression (a wave of neuronal and glial depolarisation, followed by long-lasting suppression of neural activity) as described in animals [14] is likely to be the experimental homologue of the migraine aura. Cortical spreading depression and migraine aura share many features, and recent demonstrations of phenomena similar to cortical spreading depression during aura in patients are convincing [15].

Do Granziera and colleagues' new data provide more information about aura? The authors believe the changes may have been caused by aura, and by inference suggest that because patients with migraine without aura have the same changes, they have clinically silent aura. An alternative view would be that the inherited basis for migraine is responsible for a developmental change that leads to the structural differences, and has no relationship to aura. Motion sensitivity is as marked in children with migraine as adults with migraine, and there are no longitudinal data examining whether the structural changes progress with time. The authors' silent aura hypothesis would predict increasing change with age, especially with attack frequency, while the alternative trait hypothesis presented here would predict a static defect. More research will provide the answer, and certainly the question is tractable.

## Conclusion

Is there more change in the migrainous brain than we have previously thought? I think the new data show that after four millennia, migraine still has many more secrets to be uncovered. A common disorder such as migraine needs extensive and in-depth study. Brain imaging is an important part of that work. For patients and physicians alike, the new data certainly plant migraine firmly in the brain in terms of the fundamental problem and its most crucial manifestations. The neurobiology of migraine is complex and its study rewarding.
